# Prediction of maturity-onset diabetes of the young subtypes using machine learning

**DOI:** 10.3389/fdgth.2026.1656161

**Published:** 2026-03-26

**Authors:** Israel Figueroa, Ricardo Flores, Andrea Millán, Alejandro de Dios, Gustavo Daniel Frechtel, Ariel Pablo López, Daniela Mennickent

**Affiliations:** 1 Facultad de Ingeniería, Universidad Católica de la Santísima Concepción, Concepción, Chile; 2 Departamento de Electrónica e Informática, Universidad Técnica Federico Santa María, Concepción, Chile; 3 Departamento de Ingeniería Informática y Ciencias de la Computación, Facultad de Ingeniería, Universidad de Concepción, Concepción, Chile; 4 Cátedra de Genética, Facultad de Farmacia y Bioquímica, Universidad de Buenos Aires, Buenos Aires, Argentina; 5 División Nutrición, Hospital de Clínicas, Facultad de Medicina, Universidad de Buenos Aires, Buenos Aires, Argentina; 6 Departamento de Ciencias Básicas y Morfología, Facultad de Medicina, Universidad Católica de la Santísima Concepción, Concepción, Chile

**Keywords:** classification, diabetes, explainable AI, machine learning, MODY, subtypes

## Abstract

**Introduction:**

Maturity-onset diabetes of the young (MODY) is a monogenic type of diabetes caused by different pathogenic genetic variants in glucose metabolism-related genes, with GCK-MODY and HFN1A-MODY subtypes being the most frequent. Diagnosing the specific MODY subtype is essential for correct treatment and follow-up, but it requires gene sequencing, a time-consuming and costly process that depends on highly skilled professionals. Therefore, it is mandatory to develop tools that allow to correctly determine in which order to study the involved genes, reducing the number of sequencing procedures to find the causal variant and making the diagnostic process more efficient. This proof-of-concept study evaluates machine learning as a complement to clinical characterization and genetic testing, by optimizing binary classification models for explainable prediction of MODY subtypes, with a focus on GCK-MODY and HFN1A-MODY.

**Methods:**

To meet this aim, we analyzed medical data from a diabetes cohort from Buenos Aires, Argentina. By employing imputation and oversampling techniques we created 10 datasets for each subtype to feed a pipeline that trained, optimized and evaluated 10 machine learning techniques.

**Results:**

Gaussian Naive Bayes achieved the best predictive power for GCK-MODY with a ROC AUC score of 0.724, meanwhile Random Forest yielded 0.712 for HNF1A-MODY. SHAP analysis provided insights into feature importance, highlighting the explainability of our approach.

**Discussion and conclusion:**

This novel study demonstrates for the first time the viability of machine learning as a supplementary tool prior to MODY genetic testing, by providing cost-effective and explainable models able to assist health professionals in the diagnosis of MODY subtypes.

## 1 Introduction

Diabetes mellitus (DM) represents a complex and heterogeneous group of metabolic disorders marked by chronic hyperglycemia due to defects in insulin secretion, insulin action, or both. This multifaceted condition manifests in various phenotypic forms, ranging from autoimmune-mediated type 1 diabetes (T1DM) to the insulin resistance-dominated type 2 diabetes (T2DM) [1, 2]. Beyond these classifications, atypical and hybrid forms challenge conventional diagnostic frameworks, contributing to diagnostic uncertainty and misclassification [3, 4]. Management encompasses pharmacological interventions such as insulin therapy, oral antihyperglycemic drugs and/or non-pharmacological strategies, including dietary modifications and structured physical activity, tailored to the individual patient’s needs [5–8].

Maturity-onset diabetes of the young (MODY) is a monogenic type of diabetes that accounts for approximately 1%–5% of all diabetes cases [3], with data suggesting that prevalence varies by country and remains poorly defined in several regions worldwide [9, 10]. It is characterized by onset at a young age, typically before 25 years, and an autosomal dominant inheritance pattern [11–13]. Despite these defining features, MODY is frequently underdiagnosed or misclassified as T1DM or T2DM, with estimates suggesting that up to 80% of cases are incorrectly classified [3, 14]. Genetic testing remains the gold standard for confirming a MODY diagnosis; however, its accessibility is limited by high costs, time requirements, and the need for specialized molecular diagnostics expertise [8, 15, 16].

To date, 14 monogenic subtypes of MODY have been described, with GCK-MODY and HNF1A-MODY being the most prevalent. Each subtype is associated with pathogenic variants in a specific gene, relatively characteristic clinical features, and distinct treatment requirements [3, 17, 18]. While certain MODY forms may only require lifestyle modifications, others may benefit from the use of particular pharmacological interventions [19–22]. However, the clinical presentation of MODY is highly heterogeneous and often overlaps with other forms of diabetes, complicating clinical recognition [23, 24]. For example, HNF1A-MODY patients are frequently misdiagnosed as T1DM, while GCK-MODY patients are often classified as having T2DM [25]. Such misclassifications may lead to unnecessary or inappropriate treatment, increased healthcare costs, and avoidable complications [26, 27]. In this context, timely and accurate genetic diagnosis is essential to enable precision treatment and improve long-term outcomes [28, 29].

The diagnostic process for MODY involves the analysis of regulatory and coding regions of the 14 known MODY-related genes to identify pathogenic variants. Although the number of genes to be investigated is limited, the number of potential mutations within each gene is substantial, making comprehensive sequencing indispensable for accurate diagnosis. In clinical practice, Sanger sequencing is often employed in a sequential, gene-by-gene approach, prioritizing the gene most likely implicated based on the patient’s clinical profile. However, due to the overlapping and ambiguous nature of MODY phenotypes, this process is frequently inefficient, time-consuming, and costly [10, 30, 31]. These challenges are exacerbated in low and middle-income countries, including much of Latin America, where access to genetic testing is constrained by financial, infrastructural, and logistical limitations [32, 33]. Moreover, uncertainty regarding pathogenicity assessment, particularly for rare or low-penetrant variants, further complicates diagnostic interpretation [10, 34, 35].

In recent years, machine learning has emerged as a promising strategy to support MODY identification by leveraging routinely collected clinical data to guide genetic testing more efficiently. Moreover, the development of explainable machine learning models may allow for interpretable predictions [36], enabling health professionals to better understand the model’s results, and thus to make more informed clinical decisions in a cost-effective manner. Statistical and computational models, such as the University of Exeter MODY probability calculator, have demonstrated improved discrimination between MODY and other diabetes types and increased diagnostic yield in selected populations [37–39]. However, these models were largely developed in White European cohorts and may not generalize optimally to ethnically diverse populations, where differences in phenotype distribution and prevalence of young-onset T2DM are evident [33, 40, 41]. Importantly, existing tools focus on identifying MODY as a broad category and do not address the classification of individual MODY subtypes.

This gap motivates the present study, which aims to evaluate whether explainable machine learning models trained on routinely collected clinical data can predict specific MODY subtypes with meaningful performance. We hypothesize that such models can discriminate between the presence and absence of individual MODY subtypes better than chance while providing interpretable insights into the clinical variables driving these predictions. To this end, we propose a modular proof-of-concept pipeline that frames each MODY subtype as an independent binary classification task, enabling flexible, subtype-specific, and cost-effective clinical evaluation.

## 2 Methods

### 2.1 Ethical aspects

This study was approved by the Ethics Committee of the Hospital de Clínicas José de San Martín, Facultad de Medicina, Universidad de Buenos Aires, Buenos Aires, Argentina, and was performed in accordance to the World Medical Association Declaration of Helsinki. Participants provided written informed consent to be included in the study.

The informed consent process was carried out during the clinical consultation by the treating physician. Prior to signing the consent form, patients were provided with an information sheet describing in detail the objectives of the study, the type of data collected, the nature of their participation, and the voluntary and confidential character of the research. Patients were allowed to take this information sheet home and consider their participation without coercion. Data were anonymized prior to analysis, and participants retained the right to withdraw from the study at any time. Clinical care was provided according to standard medical practice and was not affected by the patient’s decision to participate or not in the study.

### 2.2 Dataset

The dataset used in this study was derived from the anonymized medical records of 520 persons with diabetes treated at Hospital de Clínicas José de San Martín. From this cohort, 222 patients underwent Sanger sequencing for GCK-MODY, and 122 for HNF1A-MODY, confirming or discarding the respective diagnosis. In addition, 4 subjects were tested for HNF4A-MODY or HNF1B-MODY, however they were excluded from further analyses due to the very limited sample size. Only records with confirmed Sanger test results for GCK-MODY or HNF1A-MODY were retained, while all remaining entries were discarded.

Approximately 39% of the dataset consisted of missing values. To address this problem and maximize data usability for machine learning modelling, different imputation strategies were implemented, as detailed in Section 2.4.1.1.

#### 2.2.1 Predictive variables

The dataset comprised 7 variables for prediction, obtained from clinical interviews, physical examination, and routine enzymatic blood laboratory assays. These data were recorded on standard clinical practice by physicians specialized in MODY management during patient assessment and follow-up. These included two binary features (sex and family history of diabetes) and five numerical variables (body mass index —BMI—, blood glycosylated hemoglobin —HbA1c— levels, age at diagnosis, fasting blood glucose levels, and post-load blood glucose levels 120 min after the consumption of 75 g of glucose). These variables are routinely used in the medical practice of Hospital de Clínicas José de San Martín to distinguish persons with MODY from persons with other diabetes phenotypes or from persons without diabetes, and to partially guide medical suspicion regarding which persons are candidates for specific pathogenic variants in MODY-related genes. The selection of these 7 variables was based on prior clinical knowledge of their association with certain cases of GCK-MODY and HNF1A-MODY, as well as on their availability in the hospital records.

#### 2.2.2 Target variables

Given that MODY variants are not mutually exclusive, binary classification models were trained separately for each target class. Therefore, the dataset was partitioned based on Sanger sequencing results to create distinct subsets for GCK-MODY (162 positive and 60 negative) and HNF1A-MODY (48 positive and 74 negative).

### 2.3 Exploratory statistical analysis

Univariate analyses were performed to assess differences between the MODY-positive and MODY-negative groups across clinical and biochemical variables. For this purpose, the analyses were conducted using the original data, without applying any preprocessing steps. Continuous variables were first tested for normality using the Shapiro-Wilk test. If normally distributed, comparisons were made using the *t*-test and summarized with mean and standard deviation; otherwise, the Mann-Whitney U test was used, reporting median and interquartile range. For categorical variables, group differences were evaluated using Fisher’s exact test, with frequencies and percentages reported. Statistical significance was set at p<0.05.

In addition to the statistical analysis, the pipeline included the generation of histograms, count plots, and boxplots for selected variables, grouped by class. A global histogram grid was produced to visualize the distribution of all features through density plots. A heatmap of the correlation matrix was computed using Pearson correlation coefficients, and principal component analysis was performed after autoscaling the data as a pretreatment step.

### 2.4 Machine learning pipeline

A comprehensive machine learning workflow was designed and implemented to address a binary classification task, with the aim of systematically evaluating the impact of different methodological choices on predictive performance. The pipeline encompassed data preprocessing, multiple classification techniques, and model optimization and evaluation, including hyperparameter tuning, validation, and performance assessment. To ensure robustness and to assess the sensitivity of the models to data characteristics, both imputed and non-imputed datasets were analyzed, as well as balanced and imbalanced versions of the data. This strategy enabled a controlled comparison of multiple machine learning algorithms with heterogeneous assumptions and modeling capacities, providing a broad perspective on their suitability for the problem under investigation.

#### 2.4.1 Data preprocessing

##### 2.4.1.1 Missing values and class imbalance

Missing values were handled using two imputation methods: Multivariate Imputation by Chained Equations (MICE) [42] and KNN Imputer [43]. To evaluate the impact of missing values, we created three levels of datasets:

- Zero Imputation: Using only records with no missing values.
- Half Impute: Including records with fewer than three missing variables.
- Full Dataset: Incorporating all records with imputed values.

For datasets with half and full imputation, both MICE and KNN Imputer methods were evaluated. To address class imbalance, oversampling dataset was additionally evaluated. The oversampling was applied only for the training split. In total, five base datasets (one original and four imputed) were generated, each with and without oversampling, resulting in ten datasets used for model evaluation.

##### 2.4.1.2 Data splitting and normalization

Data preprocessing included 80/20 training-validation split, stratified by class to preserve target proportions, and feature standardization using StandardScaler to ensure zero mean and unit variance on training data for both binary and numerical variables.

#### 2.4.2 Evaluated classification techniques

The assessed algorithms can be broadly categorized into traditional parametric models and more flexible non-parametric approaches. The parametric models assume an explicit functional form linking the input features to the class membership probability. This group includes Logistic Regression, which models the probability of the positive class through a sigmoid transformation of a linear predictor; the Perceptron and the Stochastic Gradient Descent Classifier (SGD), which iteratively optimize linear decision functions by minimizing a specified loss function; Linear Discriminant Analysis (LDA), which derives linear decision boundaries under the assumption of Gaussian class-conditional distributions with shared covariance matrices; and Partial Least Squares Discriminant Analysis (PLS-DA), which projects the predictors onto a latent space that maximizes covariance with the response variable. In contrast, non-parametric or semi-parametric models impose fewer assumptions on the data-generating process and are capable of capturing complex non-linear relationships. These include Support Vector Machine (SVC) with kernel functions, which construct optimal separating hyperplanes in high-dimensional feature spaces; K-Nearest Neighbors (KNeighbors), which performs classification based on local similarity among observations; Gaussian Naive Bayes (GaussianNB), which combines simple probabilistic assumptions with empirical estimates of feature distributions; and ensemble-based methods such as Random Forest and XGBClassifier (XGBoost), which aggregate multiple decision trees to enhance predictive accuracy and generalization. Together, these 10 complementary modeling paradigms enabled a comprehensive assessment of classification performance across a wide range of statistical assumptions and learning capacities.

#### 2.4.3 Model optimization and evaluation

For each machine learning algorithm, a hyperparameter search space was defined (Supplementary Table S1) and systematically explored using grid search implemented via Ray Tune in combination with GridSearchCV. The model configuration achieving the highest Receiver Operating Characteristic Area Under the Curve (ROC AUC) on the training data was identified as the best-performing model. This model was then evaluated on the validation split to compute performance metrics, including ROC AUC, sensitivity, specificity, positive predictive value (PPV), negative predictive value (NPV), and Brier score, which were retained for subsequent analysis. Model validation was performed using bootstrap resampling to generate multiple randomized training and validation splits, enhancing robustness against data variability. For each bootstrap iteration, a hyperparameter search was conducted. Finally, the metrics were averaged across bootstrap iterations to obtain stable and unbiased performance estimates.

### 2.5 Explainability

To provide the explainability of machine learning models, SHapley Additive exPlanations (SHAP) values [44] were employed. SHAP is a unified framework based on cooperative game theory that assigns each feature a consistent and locally accurate importance value for individual predictions. Unlike other explainability techniques, SHAP is model-agnostic, allowing its application to any machine learning model regardless of its internal architecture or training process, thus enabling robust and interpretable explanations across different classifiers.

### 2.6 Software

The pipeline was implemented in Python (version 3.10) and executed within a Miniconda environment. The main libraries used include scikit-learn, shap, imbalanced-learn, xgboost, and ray-tune. To facilitate reproducibility, the complete codebase, environment specifications, and anonymized datasets required to rerun the same experiments are publicly available at https://github.com/ifiguero/mody_2024.

## 3 Results

### 3.1 GCK-MODY dataset

#### 3.1.1 Univariate and preliminary analysis

Univariate analyses of the zero-imputation GCK-MODY dataset are summarized in Table 1A and provide an initial characterization of differences between diagnostic groups. Three metabolic variables—fasting glucose, post-load glucose, and HbA1c—were significantly associated with diagnostic status, with individuals carrying a positive GCK-MODY diagnosis exhibiting higher mean values across all three parameters. These findings establish a baseline description of the variables most strongly linked to diagnostic outcomes and motivate further exploratory analyses aimed at understanding how these differences manifest across the data.

**Table 1:** Characteristics of the study groups.

Variable	Unit	Negative (*n* = 30)	Positive (*n* = 43)	*p* value	Total (*n* = 73)
(A) GCK-MODY positive and negative patients					
Age at diagnosis	years	14.5 (11.0–30.0)	12.0 (10.0–20.5)	0.085 (NS)	13.0 (10.0–24.0)
Body mass index	Kg/m^2^	20.8 ± 3.5	20.1 ± 3.1	0.372 (NS)	20.4 ± 3.2
Fasting glucose	mg/dL	113 (100–117)	119 (114–126)	0.011 (*)	115 (109–124)
Post-load glucose	mg/dL	137 (119–150)	147 (136–160)	0.037 (*)	144 (131–158)
HbA1c	%	5.9 ± 0.6	6.2 ± 0.4	0.027 (*)	6.1 ± 0.5
Sex (Female)	%	60.0 (18/30)	62.8 (27/43)	0.812 (NS)	61.6 (45/73)
(Male)	%	40.0 (12/30)	37.2 (16/43)		38.4 (28/73)
Family history	%	90.0 (27/30)	97.7 (42/43)	0.299 (NS)	94.5 (69/73)
Variable	Unit	Negative (*n* = 19)	Positive (*n* = 7)	*p* value	Total (*n* = 26)
(B) HNF1A-MODY positive and negative patients					
Age at diagnosis	years	23.0 (12.0–29.0)	11.0 (11.0–19.0)	0.132 (NS)	19.0 (11.0–26.0)
Body mass index	Kg/m^2^	20.9 (18.0–23.0)	19.2 (18.0–22.1)	0.977 (NS)	19.6 (18.0–23.0)
Fasting glucose	mg/dL	111.2 ± 18.4	106.0 ± 15.1	0.476 (NS)	109.8 ± 17.5
Post-load glucose	mg/dL	198.2 ± 66.3	268.4 ± 78.6	0.063 (NS)	217.1 ± 75.2
HbA1c	%	6.1 (5.7–6.5)	6.6 (5.9–6.7)	0.469 (NS)	6.2 (5.7–6.7)
Sex (Female)	%	68.4 (13/19)	42.9 (3/7)	0.369 (NS)	61.5 (16/26)
(Male)	%	31.6 (6/19)	57.1 (4/7)		38.5 (10/26)
Family history	%	100.0 (19/19)	85.7 (6/7)	0.269 (NS)	96.2 (25/26)

Continuous variables with normal distribution are presented as mean + standard deviation. Continuous variables with non-normal distribution are presented as median (interquartile range). Categorical variables are presented as percentage (proportion). *: *p* < 0.05. NS: not significant.

To visually assess these univariate associations, boxplots (Supplementary Figures S1A–S2A) were first examined, revealing observable shifts in central tendency between diagnostic classes for several continuous predictors in the Full dataset. These patterns were complemented by distribution plots (Supplementary Figure S3A), which further illustrated differences in the overall shape and spread of both continuous and binary variables across groups. Despite these visible shifts, substantial overlap between distributions was consistently observed, indicating that individual variables, when considered in isolation, provide limited discriminative power for separating diagnostic classes.

**Figure 1:**
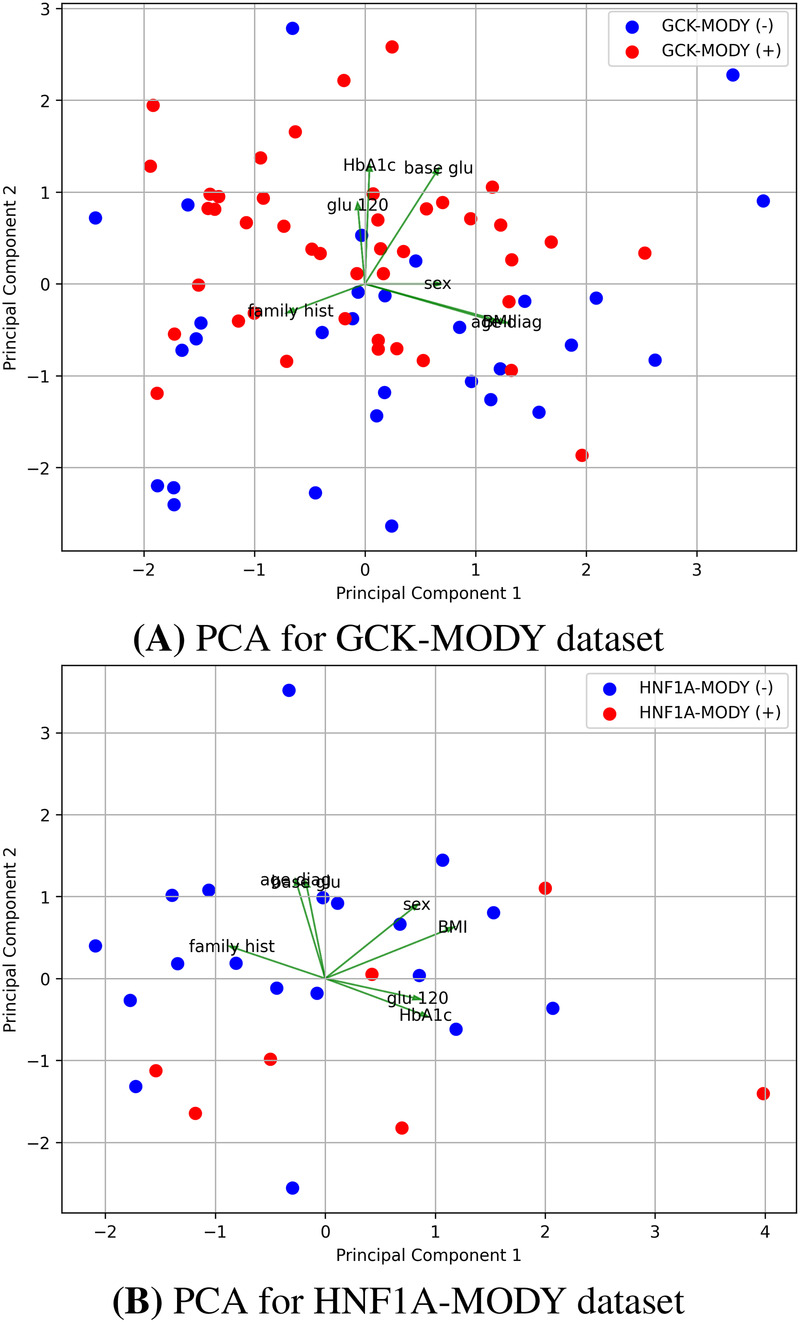
Principal Component Analysis (PCA) of MODY datasets. **(A)** PCA of the GCK-MODY dataset and **(B)** PCA of the HNF1A-MODY dataset. Each point represents an individual sample projected onto the first two principal components. Samples with pathogenic variants confirmed by Sanger sequencing are shown in red, while samples testing negative are shown in blue. Variable loadings corresponding to the input features are overlaid, illustrating their contributions to the principal component space. *BMI* corresponds to body mass index, *base glu* to fasting blood glucose levels, *glu 120* to post-load blood glucose levels 120 min after the consumption of 75 g of glucose.

Building on these observations, correlation maps (Supplementary Figure S4A) were used to examine relationships among predictors and their association with diagnostic status. Age at diagnosis showed negative correlations with most variables, whereas family history of diabetes, HbA1c, fasting glucose, and post-load glucose were positively correlated with a positive GCK-MODY diagnosis, reinforcing the trends identified in the univariate analyses. Finally, principal component analysis biplots (Figure 1A) provided a multivariate overview of the data structure. While some tendency toward clustering by diagnostic status was apparent, considerable intra-class dispersion and partial overlap between groups persisted. Collectively, these results highlight a complex and partially overlapping feature space, underscoring the need for multivariate and machine learning approaches to effectively address the classification task.

#### 3.1.2 Evaluation of machine learning models

A total of 2,500 machine learning models were trained and evaluated for GCK-MODY prediction. Performance metrics are summarized in Table 2A for ROC AUC and in Supplementary Tables S2A–S6A for sensitivity, specificity, PPV, NPV, and Brier score. The final hyperparameter configurations for the best-performing models are reported in Supplementary Table S7.

**Table 2:** Average ROC AUC scores for the classification models.

	Zero		Half KNN		Half MICE		Full KNN		Full MICE	
Model	unb	ovr	unb	ovr	unb	ovr	unb	ovr	unb	ovr
(A) Performance (ROC AUC) for the GCK-MODY dataset										
GaussianNB	0.653	0.664	0.654	0.671	0.656	0.674	**0.652**	**0.682**	**0.702**	**0.724**
KNeighbors	0.589	0.630	0.649	**0.673**	0.625	0.639	0.593	0.661	0.612	0.678
LDA	0.639	**0.687**	0.663	0.667	**0.686**	0.672	0.592	0.612	0.623	0.665
Logistic regression	0.648	0.669	**0.666**	0.664	0.661	**0.680**	0.577	0.609	0.603	0.670
Perceptron	0.618	0.642	0.616	0.665	0.618	0.592	0.534	0.522	0.602	0.558
PLS DA	**0.657**	**0.687**	0.656	0.657	0.673	0.667	0.580	0.608	0.577	0.654
Random forest	0.611	0.620	0.665	0.667	0.674	0.679	0.630	0.662	0.670	0.676
SGD	0.653	0.626	0.663	0.629	0.674	0.670	0.568	0.617	0.613	0.656
SVC	0.651	0.669	0.650	0.655	0.661	0.668	0.650	0.631	0.654	0.669
XGBoost	0.600	0.620	0.624	0.585	0.631	0.636	0.612	0.639	0.637	0.658
(B) Performance (ROC AUC) for the HNF1A-MODY dataset										
GaussianNB	**0.515**	0.510	0.524	0.573	0.517	0.554	0.591	0.579	0.625	0.619
KNeighbors	0.480	0.550	**0.576**	**0.628**	**0.559**	**0.587**	0.563	0.531	0.632	0.623
LDA	0.485	0.550	0.506	0.588	0.511	0.585	0.482	0.537	0.463	0.549
Logistic regression	0.500	0.510	0.497	0.581	0.492	0.579	0.481	0.539	0.487	0.545
Perceptron	0.505	0.555	0.550	0.512	0.549	0.528	0.507	0.540	0.478	0.468
PLS DA	0.490	0.540	0.511	0.603	0.496	0.583	0.485	0.541	0.461	0.555
Random forest	0.440	**0.565**	0.525	0.568	0.543	0.551	**0.619**	**0.639**	**0.712**	**0.703**
SGD	0.475	0.545	0.496	0.560	0.529	0.529	0.467	0.541	0.479	0.569
SVC	0.505	0.465	0.489	0.530	0.490	0.528	0.526	0.590	0.602	0.627
XGBoost	0.495	0.545	0.504	0.562	0.498	0.560	0.589	0.600	0.665	0.673

For each dataset (Zero, Half KNN, Half MICE, Full KNN and Full MICE), two separate columns are shown: one for the original unbalanced sample (unb) and the other for the oversampled data (ovr). Bold indicates the best score for the sample, and underlining indicates the best score for the technique.

GaussianNB classifier achieved the highest predictive performance, with a ROC AUC of 0.724, using the dataset processed with full MICE imputation and oversampling. This result reflects moderate discrimination ability. KNeighbors, LDA, logistic regression, and PLS-DA models achieved comparable performance. In contrast, the XGBoost yielded the lowest predictive power, with a ROC AUC of 0.658 under the same preprocessing conditions.

Across models, oversampled datasets consistently outperformed their unbalanced counterparts, and MICE-based imputations generally resulted in better performance than KNN imputations. Calibration analysis based on the Brier score showed that GaussianNB exhibited improved calibration when trained on oversampled data, whereas oversampling led to increased miscalibration for all other models.

The best-performing GCK-MODY model achieved a sensitivity of 0.885 and a specificity of 0.570, with a PPV of 0.853 and an NPV of 0.656. These results indicate good ability to correctly identify true GCK-MODY cases, albeit at the expense of limited performance in correctly identifying true negatives.

Overall, these results indicate that machine learning models trained on routinely collected clinical variables can discriminate GCK-MODY cases from non-cases better than chance, albeit with moderate accuracy. The observed performance supports the feasibility of predicting GCK-MODY subtypes.

#### 3.1.3 Models’ explainability according to SHAP values

**Figure 2:**
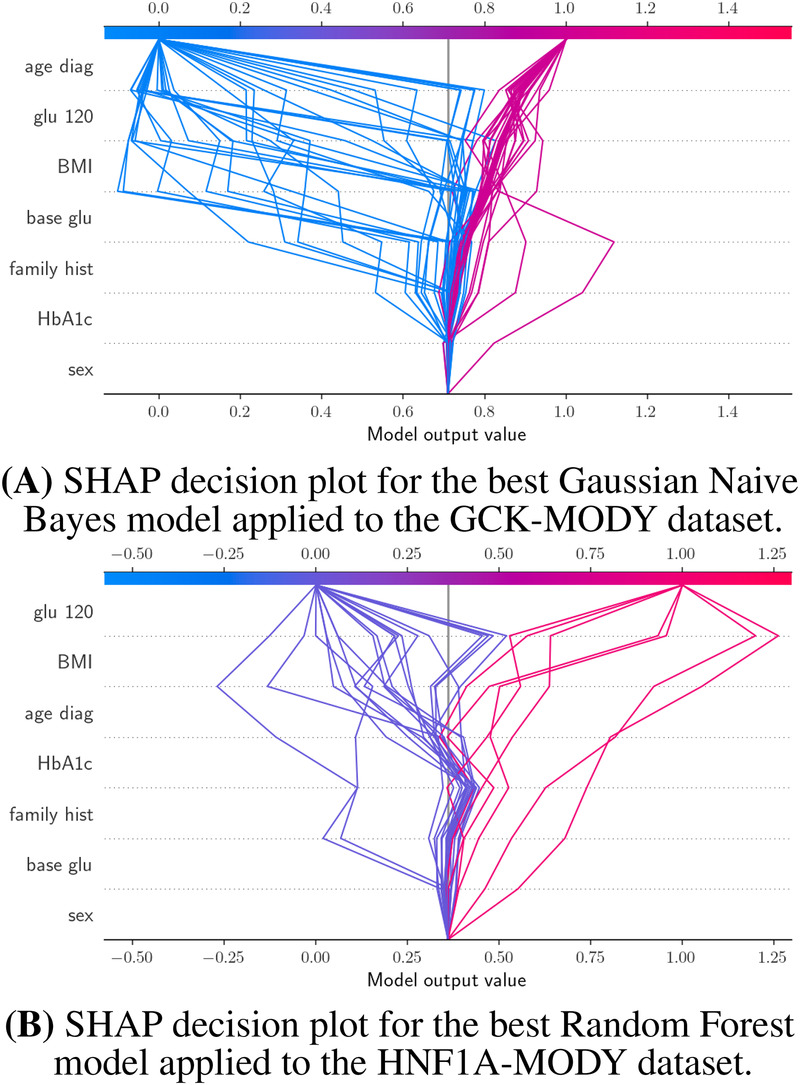
SHAP decision plots illustrating feature contributions to model predictions. **(A)** Gaussian Naive Bayes model trained on the GCK-MODY dataset and **(B)** Random Forest model trained on the HNF1A-MODY dataset. Each line represents an individual subject, with model predictions starting from the expected value at the bottom of the plot. Rows correspond to input variables, and horizontal shifts indicate the contribution of each variable to the final prediction. Lines colored in red correspond to positive class predictions (class 1), while lines in blue correspond to negative class predictions (class 0). *BMI* corresponds to body mass index, *base glu* to fasting blood glucose levels, *glu 120* to post-load blood glucose levels 120 min after the consumption of 75 g of glucose.

To interpret the predictions of the best-performing GCK-MODY model, SHAP-based explainability analyses were conducted. Figure 2A summarizes the overall contribution of each variable to the model’s predictions. age at diagnosis, fasting glucose and HbA1c emerged as the most influential factors driving positive GCK-MODY predictions. Conversely, fasting glucose, BMI, post-load glucose, and age at diagnosis were the primary contributors to negative predictions.

Although the relative importance of predictors varied across individual samples, the model consistently relied on variables that were also statistically significant in univariate analyses, namely fasting glucose, post-load glucose, and HbA1c. This concordance between traditional statistical analysis and model explainability supports the internal coherence of the predictive framework. Additionally, Supplementary Figure S5 illustrates SHAP explanations at the individual level, demonstrating how specific variables influenced model predictions for selected samples and enabling transparent case-by-case assessment.

Importantly, the alignment between SHAP-derived feature contributions and clinically established biomarkers of GCK-MODY indicates that the model’s predictions are driven by meaningful and interpretable clinical signals. This supports the hypothesis that explainable machine learning approaches can provide transparent insights into the variables underlying subtype-specific MODY predictions.

### 3.2 HNF1A-MODY dataset

#### 3.2.1 Univariate and preliminary analysis

Univariate analysis results for the zero-imputation HNF1A-MODY dataset are reported in Table 1B and reveal a markedly different pattern compared with the GCK-MODY cohort. None of the evaluated variables reached statistical significance at the univariate level. Nonetheless, post-load glucose and age at diagnosis exhibited trends toward association with diagnostic status, with *p*-values of 0.063 and 0.132, respectively, suggesting weak and inconclusive signals that warrant further multivariate investigation.

Univariate visualizations were subsequently examined to further characterize these patterns. Box plots of continuous variables and summaries of binary predictors (Supplementary Figures S1B–S2B) showed only modest differences in central tendency between diagnostic classes, with substantial overlap across groups. Distribution plots (Supplementary Figure S3B) reinforced this observation, indicating limited discriminative capacity when individual variables were considered in isolation. Consistent with these findings, correlation maps (Supplementary Figure S4B) revealed a structure broadly similar to that observed in the GCK-MODY analysis, with age at diagnosis negatively correlated with most predictors. In contrast to the GCK-MODY dataset, however, post-load glucose emerged as the only variable exhibiting a noticeable positive correlation with HNF1A-MODY diagnosis, underscoring a narrower set of potentially informative features.

Principal component analysis biplots (Figure 1B) further corroborated the limited separability suggested by the univariate analyses, showing pronounced overlap between diagnostic classes and an absence of well-defined clustering by disease status. Collectively, these descriptive, visual, and exploratory findings highlight the greater difficulty of class separation in the HNF1A-MODY dataset and reinforce the need for multivariate modeling approaches to capture subtle and potentially nonlinear relationships within the data.

#### 3.2.2 Evaluation of machine learning models

For HNF1A-MODY, 2,500 models were similarly trained and evaluated. Performance metrics are summarized in Table 2B and Supplementary Tables S2B–S6B. The Random Forest classifier achieved the highest ROC AUC of 0.712, corresponding to moderate classification performance. This result was obtained using the full MICE imputation with unbalanced classes, although comparable performance was observed across datasets with full imputations.

KNeighbors models achieved their best results on datasets with half imputations, while the Perceptron classifier showed the lowest overall performance, reaching a maximum ROC AUC of 0.555 on the zero-imputation oversampled dataset. These findings underscore the sensitivity of model performance to imputation strategy and class balancing. Calibration analysis revealed evidence of miscalibration across all models, which generally worsened with oversampling.

The best-performing HNF1A-MODY model achieved a sensitivity of 0.468 and a specificity of 0.757, with PPV and NPV values of 0.579 and 0.682, respectively. These metrics indicate limited ability to identify true positive cases, but a moderate capacity to correctly rule out HNF1A-MODY in negative individuals.

Overall, these findings indicate that machine learning models trained on routinely collected clinical data can discriminate HNF1A-MODY cases from non-cases better than chance, although with lower sensitivity compared to GCK-MODY. This supports the feasibility of the proposed modular framework while highlighting subtype-specific differences in achievable predictive performance.

#### 3.2.3 Models’ explainability according to SHAP values

SHAP-based explainability analysis was applied to the best-performing HNF1A-MODY model to elucidate the variables driving its predictions. As shown in Figure 2B, age at diagnosis, fasting glucose, post-load glucose, and HbA1c were the most influential variables contributing to negative predictions. In contrast, BMI, post-load glucose, and age at diagnosis had the greatest impact on positive predictions.

Even though univariate analyses did not identify statistically significant predictors for HNF1A-MODY, SHAP results indicate that the model consistently assigned substantial importance to post-load glucose and age at diagnosis. Notably, these variables exhibited trends toward statistical significance in descriptive analyses, suggesting that multivariate modeling was able to capture subtle patterns not evident in univariate testing alone.

Despite the absence of statistically significant predictors in univariate analyses, SHAP-based explanations suggest that the model relied on clinically plausible variables to inform its predictions. This indicates that explainable machine learning can uncover multivariate patterns relevant to HNF1A-MODY while maintaining transparency in how individual clinical features contribute to subtype-specific predictions.

## 4 Discussion

### 4.1 Strengths of this study

To our knowledge, this is the first study to address the prediction of MODY subtypes using clinical data and machine learning. Previous studies have explored the differentiation of MODY from other diabetes types (e.g., T1DM and T2DM) but not the classification of MODY subtypes [34, 37].

In this context, this work also provides a systematic evaluation of state-of-the-art machine learning models for generating explainable predictions in an underrepresented Latin American population. Most existing predictive tools and probability calculators for MODY have been developed and validated predominantly in European ancestry cohorts, raising concerns about their transferability across ethnic groups [33]. By focusing on a Latin American dataset, this study addresses an important gap in the literature and provides evidence that explainable machine learning approaches can be meaningfully applied in populations that are often overlooked in precision diabetes research. Expanding and diversifying available datasets remains a key priority, as larger and more representative cohorts would enable the development of more robust and generalizable models. Such efforts are essential for improving diagnostic accuracy and clinical applicability, especially given the documented limitations of existing tools when applied to non-European populations [29].

Focusing on GCK-MODY and HNF1A-MODY—the two most prevalent MODY subtypes—offers clear and clinically meaningful benefits that justify their prioritization in predictive modeling. These subtypes differ substantially in prognosis and treatment, making their accurate identification particularly impactful. GCK-MODY is typically characterized by mild, stable hyperglycemia that often does not require pharmacological intervention, whereas HNF1A-MODY is frequently misdiagnosed as T1DM and consequently treated unnecessarily with insulin [3]. Accurate identification of these subtypes therefore has immediate therapeutic implications, with the potential to improve glycemic control, quality of life, and cost-effectiveness of care.

Machine learning represents a promising approach to complement costly or invasive diagnostic procedures. However, its clinical adoption depends critically on the interpretability of model predictions. In high-stakes domains such as healthcare, clinicians must understand the rationale behind each model decision. To address this, we employed the SHAP library to enhance model explainability. To our knowledge, this is the first study to apply SHAP to assess the contribution of input features in the classification of MODY subtypes. Notably, the features identified as important by SHAP are consistent with those used in medical practice to suspect GCK-MODY and HNF1A-MODY, suggesting that the model’s reasoning aligns with established clinical knowledge. Together, the ability of machine learning models to support the identification of MODY subtypes and the integration of explainability techniques reinforces their potential role in advancing precision medicine for monogenic diabetes.

In this context, the integration of SHAP-based explainability provides novel insights into how predictive models weight clinical and biochemical variables, moving beyond black-box predictions. Unlike traditional methods that typically offer only global interpretability, SHAP enables a detailed examination of the factors driving individual predictions. This feature is particularly valuable when two patients receive the same predicted label but differ substantially in the underlying contributing variables. Moreover, SHAP facilitates the identification of cases in which the model prediction diverges from the ground truth, allowing clinicians to recognize potential misclassifications and to better understand class overlap. Together, these insights can guide iterative model refinement and foster greater clinical trust and usability of machine learning–based decision support systems.

The models developed achieved moderate predictive power for GCK-MODY and HNF1A-MODY. Based on its performance metrics, the GCK-MODY model could serve as a screening tool prior to genetic confirmation through sequencing, but not as an exclusion test, since a negative result does not fully rule out the diagnosis. In contrast, the HNF1A-MODY model—given its tendency to miss a substantial proportion of positive cases—has limited value as a screening tool. However, it may still be useful as a complementary aid in ruling out this diagnosis when combined with clinical evaluation.

Finally, the proposed pipeline was designed with scalability and flexibility in mind, enabling the integration of additional MODY subtypes, larger datasets, and new clinical or biochemical features. This modular architecture facilitates the inclusion of new predictors and supports extended model selection and hyperparameter optimization procedures.

### 4.2 Limitations and future work

The small number of available cases represents a central limitation of this study and reflects a common challenge in MODY research. These monogenic forms of diabetes are rare, costly to confirm via Sanger sequencing, and often underdiagnosed, which substantially limits data availability. Increasing the size and diversity of the dataset would likely improve model accuracy by reducing uncertainty associated with data scarcity. Although imputation techniques performed satisfactorily in handling missing values, minimizing data gaps would further enhance prediction reliability. Increasing sample size would also allow exploration of more complex artificial intelligence architectures, such as deep learning, representing a promising avenue for future research. These models require considerably larger datasets for reliable training, but may be better suited to capture intricate data patterns, potentially improving predictive accuracy and robustness.

Given the low overall prevalence of MODY (approximately 1%–5% of all diabetes cases [3]) and the even lower frequency of its individual subtypes, achieving balanced datasets for model training remains a considerable challenge. Even though balancing the training data can improve predictive accuracy, it may also introduce model miscalibration, as reflected by higher Brier scores on the oversampled dataset shown in Supplementary Table S6. Since real-world clinical data are inherently imbalanced, models trained on artificially balanced datasets may not generalize effectively to practical settings. Future work should therefore focus on developing strategies that address class imbalance while maintaining robust model calibration and real-world applicability.

Future work should also include a systematic evaluation of SHAP-based explainability outputs in collaboration with healthcare professionals. This will help validate the correspondence between model-derived feature weights and expert clinical reasoning, ensuring that the models remain interpretable, trustworthy, and aligned with real-world medical decision-making.

The proposed pipeline can also be extended to datasets including other MODY subtypes. Incorporating additional clinical and biochemical variables into the input features—such as renal or cardiovascular pathophysiological features when studying HNF4A-MODY or HNF1B-MODY—may further optimize the models’ ability to predict individual MODY subtypes. Expanding the feature set could enable the identification of more informative patterns and improve classification performance.

## 5 Conclusion

This study demonstrates the feasibility of predicting specific MODY subtypes from routinely collected medical data in patients with prior clinical suspicion of MODY using machine learning models. Despite their moderate performance and limited scope—addressing only 2 of the 14 currently known MODY subtypes—, our results highlight the potential of machine learning-based tools to assist healthcare professionals in identifying MODY subtypes more efficiently. Such tools may save valuable time and resources in the diagnostic process and support more informed decisions regarding patient management and treatment selection, particularly in settings where access to genetic testing is limited or delayed.

Taken together, the contribution of this work lies not only in establishing this proof of concept, but also in doing so through a clinically grounded and explainable machine learning framework, evaluated in an underrepresented Latin American population. By focusing on GCK-MODY and HNF1A-MODY—the two most prevalent MODY subtypes with well-established clinical relevance—and adopting a flexible, modular methodological design, this study provides a foundation that can be refined and expanded as larger and more diverse datasets become available.

## Data Availability

The datasets presented in this study can be found in online repositories. The names of the repository/repositories and accession number(s) can be found below: https://github.com/ifiguero/mody_2024.
