# Supplementary material for: Prediction of maturity-onset diabetes of the young subtypes using machine learning
Source: Front Digit Health. 2026 Mar 26;8:1656161. doi: 10.3389/fdgth.2026.1656161 (PMC13062332; doi:10.3389/fdgth.2026.1656161)
Supplement: Supplementary file 1 [file Datasheet1.pdf]

Supplementary Material

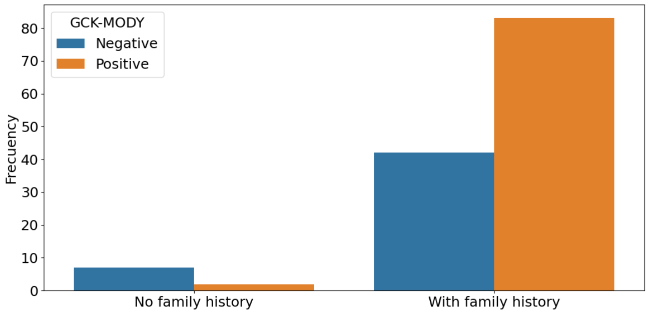

(A) Family history for GCK-MODY

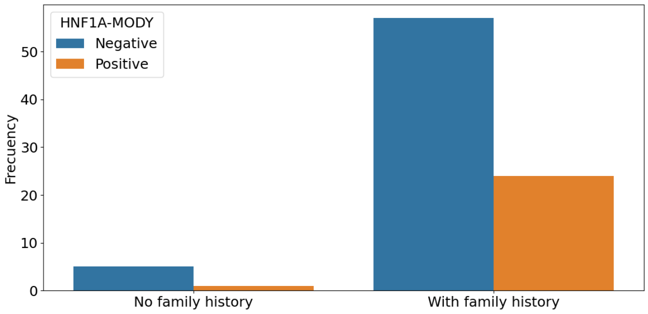

(B) Family history for HNF1A-MODY

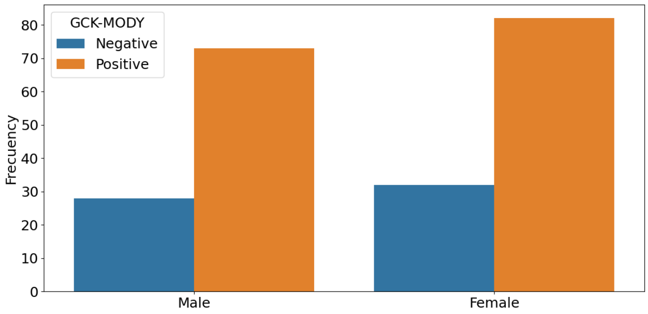

(C) Sex for GCK-MODY

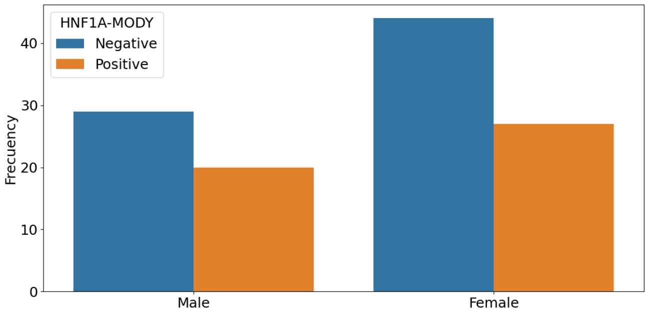

(D) Sex for HNF1A-MODY

Figure S1. Frecuency distribution for GCK-MODY and HNF1A-MODY binary variables for Full Dataset

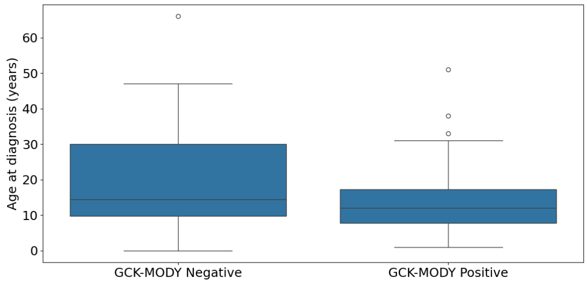

(A) Age of diagnosis for GCK-MODY

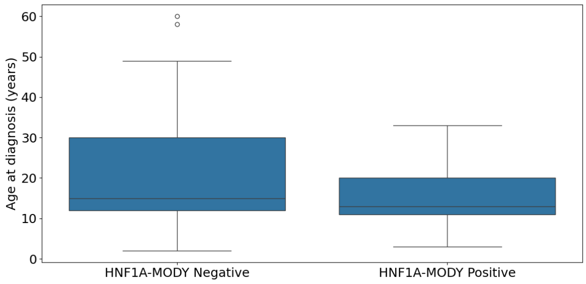

(B) Age of diagnosis for HNF1A-MODY

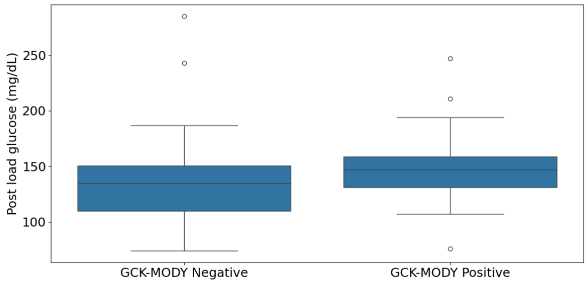

(C) Fasting Glucose for GCK-MODY

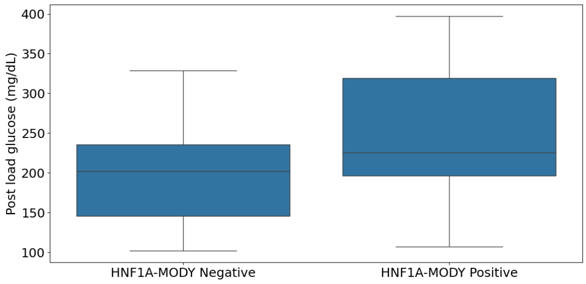

(D) Fasting Glucose for HNF1A-MODY

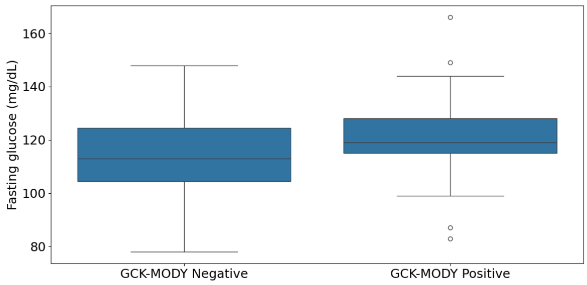

(E) Post load Glucose for GCK-MODY

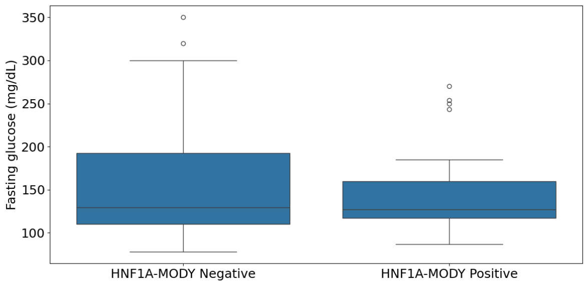

(F) Post load Glucose for HNF1A-MODY

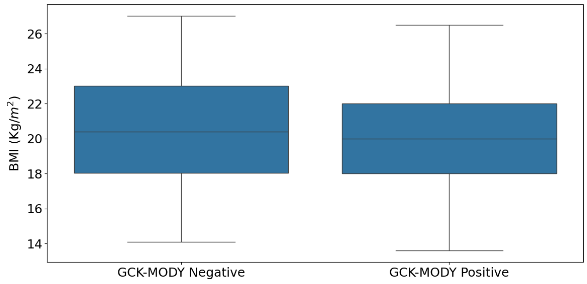

(G) Body Mass Index for GCK-MODY

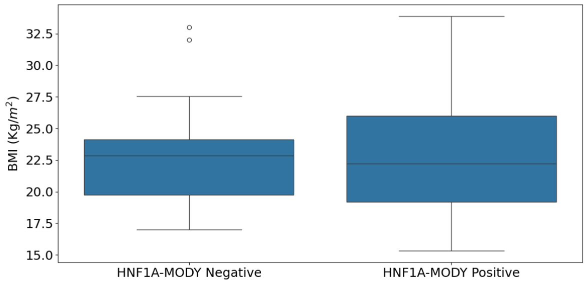

(H) Body Mass Index for HNF1A-MODY

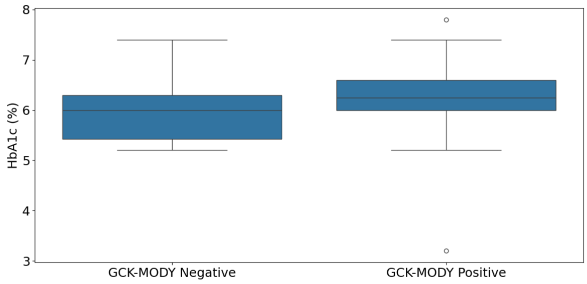

(I) HbA1c for GCK-MODY

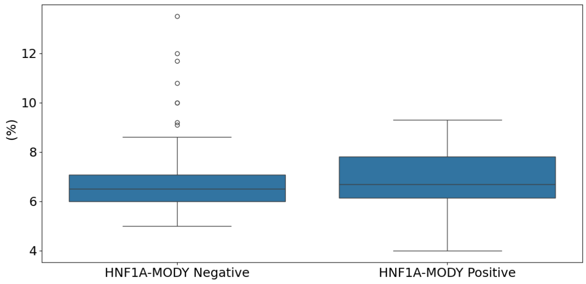

(J) HbA1c for HNF1A-MODY

Figure S2. Univariate ana1lysis comparison for GCK-MODY and HNF1A-MODY Full Dataset. Each subfigure display boxplots of given variable for both classes

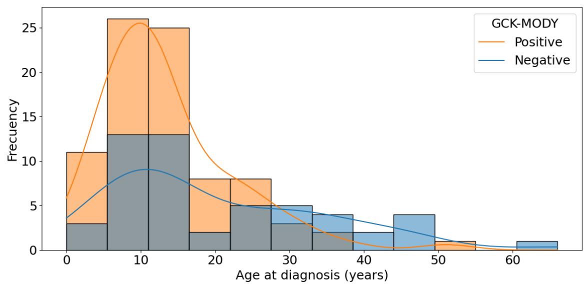

(A) Age of diagnosis for GCK-MODY

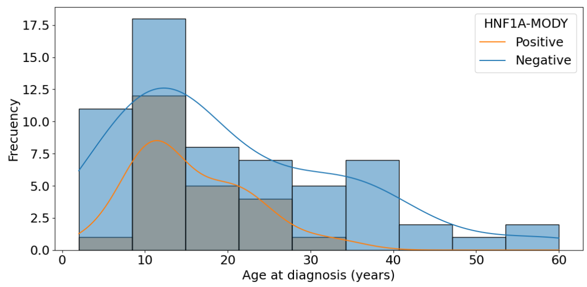

(B) Age of diagnosis for HNF1A-MODY

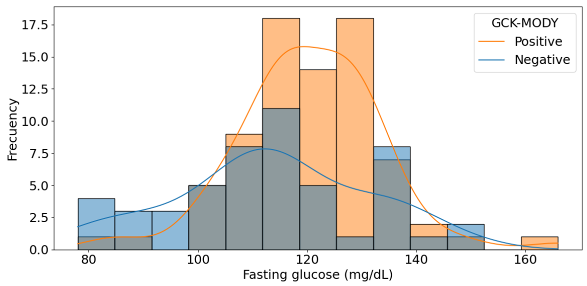

(C) Fasting Glucose for GCK-MODY

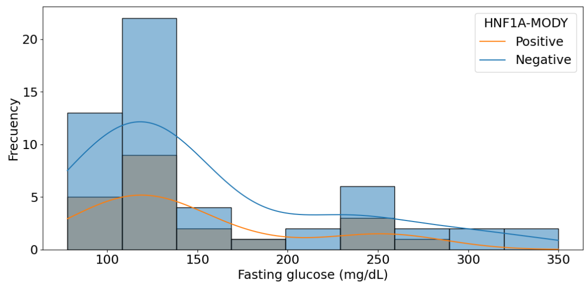

(D) Fasting Glucose for HNF1A-MODY

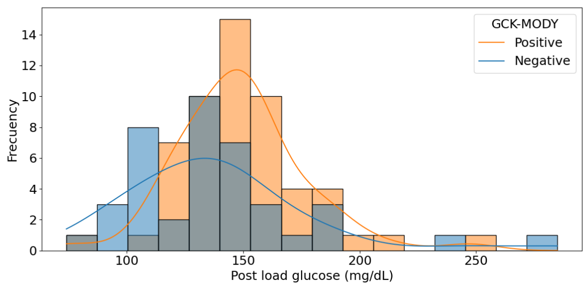

(E) Post load Glucose for GCK-MODY

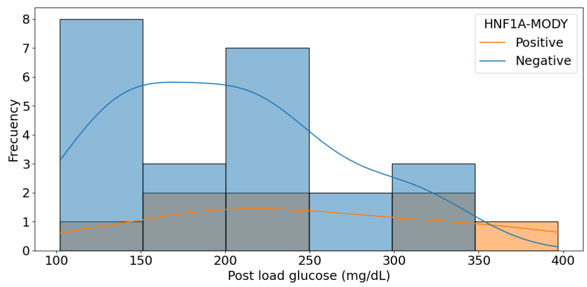

(F) Post load Glucose for HNF1A-MODY

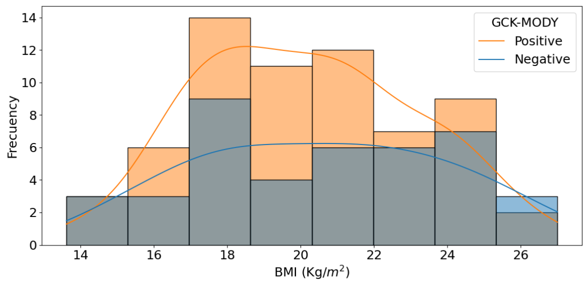

(G) Body Mass Index for GCK-MODY

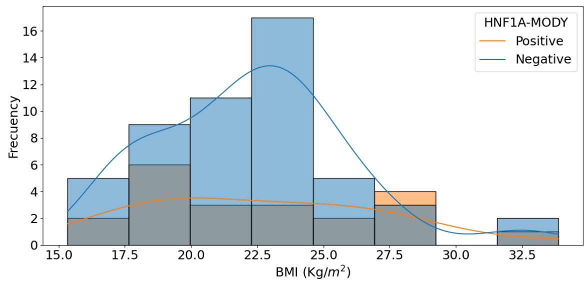

(H) Body Mass Index for HNF1A-MODY

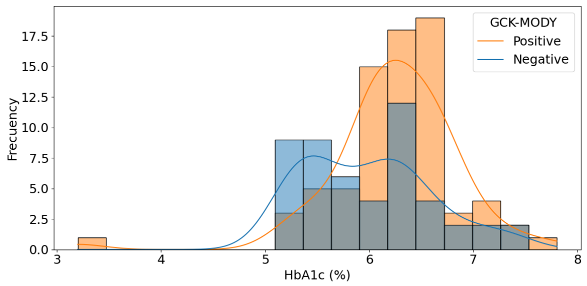

(I) HbA1c for GCK-MODY

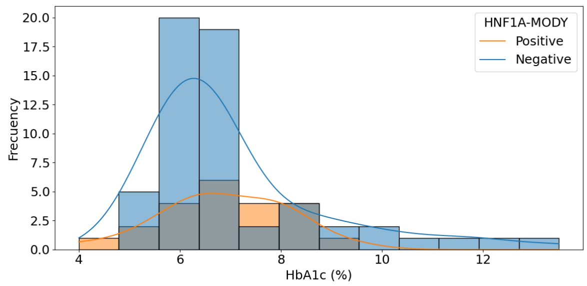

(J) HbA1c for HNF1A-MODY

Figure S3. Density plots for GCK-MODY and HNF1A-MODY Full Datasets. On each Subplot, frecuency histogram are displayed for both groups, along with their kernel density

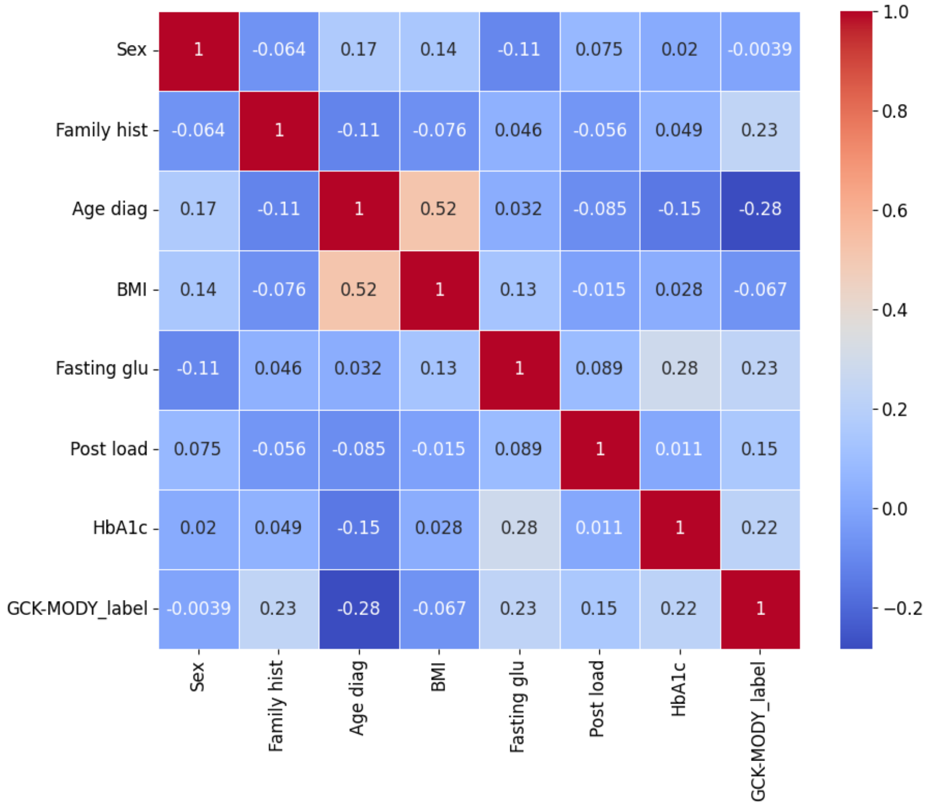

(A) Correlation Heatmap for GCK-MODY

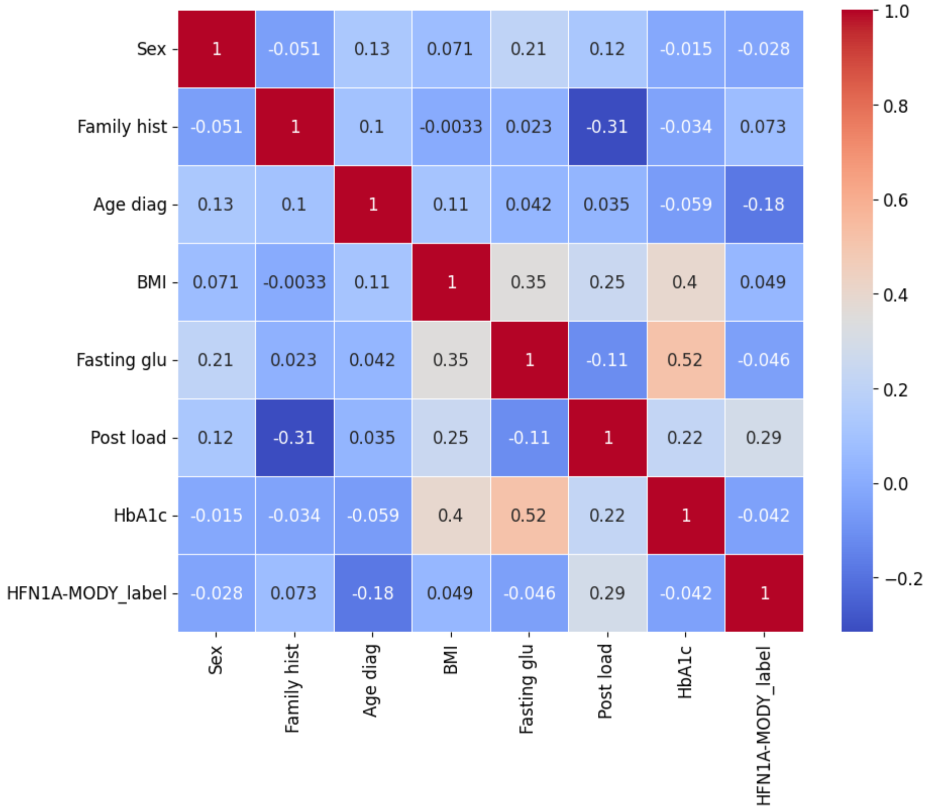

(B) Correlation Heatmap for HNF1A-MODY

Figure S4. Correlation heatmaps for the Full Dataset. Blue is negative correlation, red positive one. Darker colors displays higher correlations witithin the variables. Bottom row displays correlation with target class

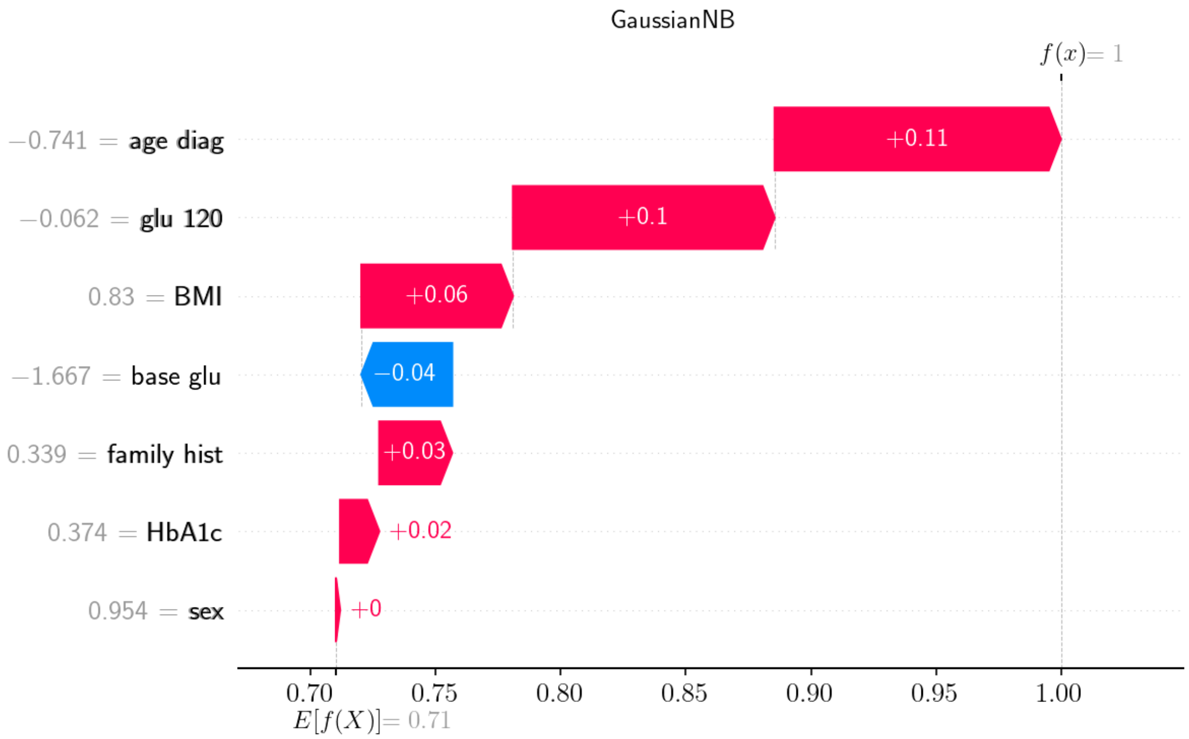

(A) Model prediction GCK-MODY positive

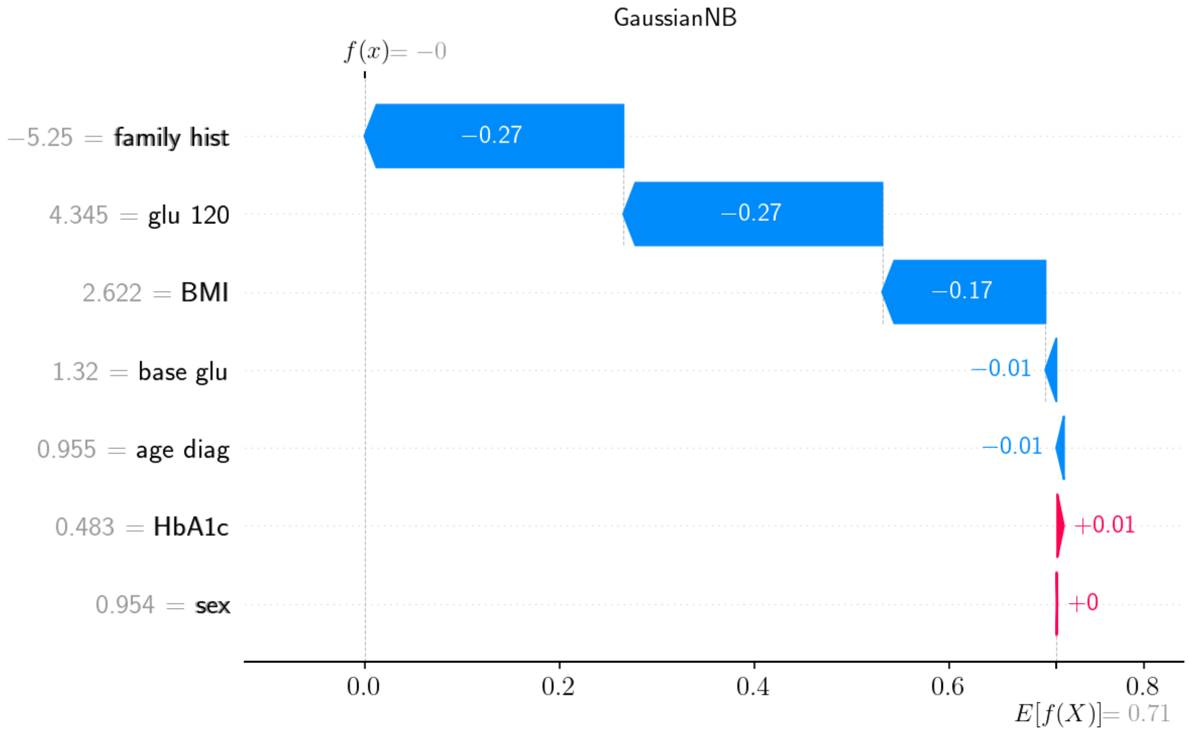

(B) Model prediction GCK-MODY negative

Figure S5. This SHAP Waterfall plot provides an overview of variable contributions to MODY class predictions for individual sample records. Starting from the base expected value, it shows how each variable influences the model, ultimately leading to a prediction of either 0 or 1

Table S1. Hyperparameters search space for models

Model	Hyperparameters seach space
LogisticRegression	$C : logspace(-2, 2, 15)$<br>$max_{iter} : [80, 100, 150]$
XGBClassifier	$n_{estimators} : [50, 100, 200]$<br>$learning_{rate} : logspace(-4, -1, 8)$<br>$max_{depth} : [3, 5, 7]$
Perceptron	$penalty : [''l2'', ''l1'', ''elasticnet'']$<br>$max_{iter} : [50, 100, 200]$
RandomForest	$n_{estimators} : [50, 100, 200]$<br>$max_{depth} : [5, 10, 15]$<br>$max_{features} : [2, 5, 10]$
SGD	$alpha : logspace(-4, -1, 8)$<br>$max_{iter} : [100, 300, 500]$<br>$penalty : [''l2'', ''l1'', ''elasticnet'']$
SVC	$C : logspace(-1, 2, 15)$<br>$kernel : [''linear'', ''poly'', ''rbf'', ''sigmoid'']$
LDA	$solver'' : [''svd'', ''lsqr'', ''eigen'']$<br>$shrinkage : [None, ''auto'']$
PLS DA	$n_{components} : [2, 3, 5]$
GaussianNB	$var_{smoothing} : logspace(-11, -8, 10)$
KNeighbors	$n_{neighbors} : [3, 5, 7, 9]$<br>$weights : [''uniform'', ''distance'']$<br>$p : [1, 2]$

Table S2. Average Sensitivity for each Dataset, two separate columns for the original unbalanced sample (unb), and with oversampling(ovr)

	Zero		Half KNN		Half MICE		Full KNN		Full MICE	
**Model**	**unb**	**ovr**	**unb**	**ovr**	**unb**	**ovr**	**unb**	**ovr**	**unb**	**ovr**
GaussianNB	0.902	0.849	0.883	0.863	0.873	0.855	0.943	0.916	0.898	0.885
KNeighbors	0.889	0.751	0.875	0.798	0.893	0.808	0.945	0.850	0.953	0.852
LDA	0.787	0.756	0.858	0.773	0.850	0.768	0.964	0.718	0.954	0.846
Logistic Regression	0.849	0.738	0.860	0.775	0.865	0.770	0.966	0.721	0.962	0.853
Perceptron	0.716	0.613	0.680	0.568	0.653	0.590	0.744	0.583	0.850	0.600
PLS DA	0.787	0.742	0.860	0.763	0.860	0.770	0.972	0.699	0.958	0.841
Random Forest	0.716	0.702	0.778	0.730	0.823	0.760	0.914	0.858	0.921	0.880
SGD	0.742	0.711	0.828	0.788	0.765	0.758	0.909	0.731	0.949	0.875
SVC	0.836	0.804	0.870	0.808	0.865	0.823	0.941	0.896	0.922	0.893
XGBoost	0.733	0.684	0.750	0.680	0.788	0.715	0.944	0.856	0.910	0.857

(A) Average Sensitivity for the models for GCK-MODY

	Zero		Half KNN		Half MICE		Full KNN		Full MICE	
**Model**	**unb**	**ovr**	**unb**	**ovr**	**unb**	**ovr**	**unb**	**ovr**	**unb**	**ovr**
GaussianNB	0.020	0.060	0.400	0.600	0.368	0.568	0.668	0.748	0.716	0.728
KNeighbors	0.020	0.420	0.224	0.504	0.144	0.544	0.596	0.688	0.532	0.604
LDA	0.100	0.340	0.032	0.488	0.056	0.512	0.304	0.600	0.088	0.448
Logistic Regression	0.020	0.360	0.008	0.496	0.000	0.512	0.208	0.596	0.004	0.440
Perceptron	0.160	0.340	0.320	0.384	0.264	0.400	0.432	0.572	0.348	0.416
PLS DA	0.100	0.340	0.048	0.504	0.040	0.528	0.316	0.612	0.100	0.456
Random Forest	0.080	0.500	0.104	0.296	0.112	0.264	0.496	0.600	0.468	0.556
SGD	0.200	0.340	0.088	0.512	0.072	0.512	0.528	0.592	0.000	0.576
SVC	0.040	0.240	0.048	0.336	0.016	0.320	0.376	0.644	0.488	0.636
XGBoost	0.000	0.340	0.056	0.328	0.016	0.288	0.468	0.624	0.472	0.604

(B) Average Sensitivity for the models for HNF1A-MODY

Table S3. Average Specificity for each Dataset, two separate columns for the original unbalanced sample (unb), and with oversampling(ovr)

	Zero		Half KNN		Half MICE		Full KNN		Full MICE	
**Model**	**unb**	**ovr**	**unb**	**ovr**	**unb**	**ovr**	**unb**	**ovr**	**unb**	**ovr**
GaussianNB	0.420	0.487	0.416	0.468	0.436	0.488	0.330	0.387	0.513	0.570
KNeighbors	0.280	0.540	0.348	0.496	0.356	0.508	0.230	0.457	0.313	0.517
LDA	0.520	0.633	0.484	0.608	0.496	0.596	0.197	0.423	0.293	0.493
Logistic Regression	0.440	0.633	0.448	0.596	0.460	0.608	0.170	0.447	0.257	0.517
Perceptron	0.433	0.607	0.512	0.556	0.560	0.596	0.323	0.457	0.403	0.537
PLS DA	0.520	0.647	0.456	0.580	0.452	0.584	0.120	0.430	0.207	0.510
Random Forest	0.580	0.587	0.516	0.564	0.544	0.612	0.413	0.483	0.470	0.523
SGD	0.540	0.620	0.464	0.560	0.504	0.568	0.223	0.383	0.243	0.430
SVC	0.507	0.573	0.424	0.444	0.456	0.520	0.327	0.410	0.367	0.407
XGBoost	0.533	0.573	0.432	0.516	0.544	0.600	0.273	0.490	0.380	0.510

(A) Average Specificity for the models for GCK-MODY

	Zero		Half KNN		Half MICE		Full KNN		Full MICE	
**Model**	**unb**	**ovr**	**unb**	**ovr**	**unb**	**ovr**	**unb**	**ovr**	**unb**	**ovr**
GaussianNB	0.970	0.960	0.663	0.483	0.663	0.500	0.512	0.451	0.549	0.515
KNeighbors	0.950	0.800	0.890	0.537	0.877	0.543	0.672	0.541	0.699	0.619
LDA	0.850	0.780	0.903	0.617	0.887	0.647	0.827	0.619	0.808	0.485
Logistic Regression	0.960	0.770	0.977	0.627	0.990	0.650	0.875	0.624	0.979	0.480
Perceptron	0.770	0.770	0.707	0.603	0.737	0.597	0.621	0.531	0.571	0.541
PLS DA	0.870	0.760	0.917	0.610	0.907	0.627	0.829	0.616	0.800	0.501
Random Forest	0.910	0.850	0.917	0.797	0.887	0.800	0.824	0.763	0.757	0.664
SGD	0.780	0.770	0.930	0.577	0.903	0.650	0.589	0.565	0.987	0.440
SVC	0.930	0.810	0.923	0.690	0.957	0.690	0.728	0.648	0.645	0.605
XGBoost	0.990	0.820	0.950	0.787	0.960	0.777	0.789	0.699	0.781	0.640

(B) Average Specificity for the models for HNF1A-MODY

Table S4. Average Positive Predictive Value for each Dataset, two separate columns for the original unbalanced sample (unb), and with oversampling(ovr)

	Zero		Half KNN		Half MICE		Full KNN		Full MICE	
**Model**	**unb**	**ovr**	**unb**	**ovr**	**unb**	**ovr**	**unb**	**ovr**	**unb**	**ovr**
GaussianNB	0.709	0.727	0.712	0.728	0.716	0.734	0.797	0.839	0.806	0.853
KNeighbors	0.654	0.715	0.684	0.722	0.693	0.729	0.772	0.793	0.813	0.830
LDA	0.717	0.764	0.730	0.760	0.732	0.752	0.768	0.788	0.775	0.823
Logistic Regression	0.702	0.755	0.713	0.755	0.719	0.759	0.763	0.782	0.783	0.832
Perceptron	0.648	0.709	0.701	0.642	0.711	0.699	0.748	0.797	0.742	0.761
PLS DA	0.716	0.763	0.717	0.743	0.715	0.747	0.753	0.769	0.774	0.826
Random Forest	0.725	0.723	0.722	0.729	0.743	0.760	0.812	0.828	0.823	0.837
SGD	0.718	0.745	0.717	0.742	0.710	0.740	0.763	0.777	0.763	0.808
SVC	0.728	0.747	0.712	0.702	0.720	0.739	0.795	0.801	0.808	0.808
XGBoost	0.713	0.711	0.681	0.695	0.740	0.742	0.782	0.804	0.824	0.831

(A) Average Positive Predictive Value for the models for GCK-MODY

	Zero		Half KNN		Half MICE		Full KNN		Full MICE	
**Model**	**unb**	**ovr**	**unb**	**ovr**	**unb**	**ovr**	**unb**	**ovr**	**unb**	**ovr**
GaussianNB	0.167	0.400	0.365	0.334	0.349	0.324	0.483	0.481	0.520	0.504
KNeighbors	0.100	0.561	0.524	0.300	0.329	0.324	0.554	0.503	0.563	0.522
LDA	0.179	0.450	0.122	0.352	0.208	0.381	0.522	0.524	0.287	0.363
Logistic Regression	0.111	0.450	0.125	0.357	0.000	0.386	0.557	0.525	0.083	0.358
Perceptron	0.208	0.413	0.277	0.255	0.333	0.305	0.406	0.452	0.318	0.353
PLS DA	0.182	0.444	0.211	0.351	0.132	0.380	0.529	0.528	0.302	0.374
Random Forest	0.267	0.626	0.386	0.387	0.276	0.324	0.680	0.640	0.579	0.538
SGD	0.261	0.425	0.286	0.332	0.209	0.397	0.506	0.492	0.000	0.403
SVC	0.167	0.377	0.144	0.314	0.140	0.289	0.486	0.554	0.474	0.528
XGBoost	0.000	0.487	0.333	0.389	0.156	0.338	0.637	0.586	0.600	0.537

(B) Average Positive Predictive Value for the models for HNF1A-MODY

Table S5. Average Negative Predictive Value for each Dataset, two separate columns for the original unbalanced sample (unb), and with oversampling(ovr)

	Zero		Half KNN		Half MICE		Full KNN		Full MICE	
**Model**	**unb**	**ovr**	**unb**	**ovr**	**unb**	**ovr**	**unb**	**ovr**	**unb**	**ovr**
GaussianNB	0.669	0.628	0.668	0.665	0.673	0.678	0.680	0.645	0.664	0.656
KNeighbors	0.610	0.605	0.644	0.610	0.683	0.633	0.680	0.540	0.739	0.586
LDA	0.624	0.637	0.698	0.642	0.693	0.631	0.672	0.380	0.704	0.590
Logistic Regression	0.661	0.634	0.697	0.639	0.708	0.641	0.657	0.424	0.722	0.591
Perceptron	0.537	0.515	0.516	0.486	0.512	0.500	0.352	0.287	0.561	0.373
PLS DA	0.631	0.645	0.693	0.619	0.694	0.634	0.586	0.373	0.647	0.599
Random Forest	0.579	0.582	0.609	0.574	0.671	0.622	0.660	0.560	0.700	0.630
SGD	0.583	0.612	0.642	0.648	0.631	0.634	0.559	0.393	0.622	0.608
SVC	0.709	0.686	0.701	0.602	0.723	0.669	0.744	0.600	0.706	0.572
XGBoost	0.571	0.551	0.536	0.502	0.615	0.575	0.665	0.576	0.619	0.578

(A) Average Negative Predictive Value for the models for GCK-MODY

	Zero		Half KNN		Half MICE		Full KNN		Full MICE	
**Model**	**unb**	**ovr**	**unb**	**ovr**	**unb**	**ovr**	**unb**	**ovr**	**unb**	**ovr**
GaussianNB	0.665	0.673	0.683	0.706	0.689	0.711	0.697	0.724	0.738	0.736
KNeighbors	0.659	0.746	0.736	0.727	0.711	0.752	0.718	0.732	0.700	0.707
LDA	0.655	0.711	0.690	0.748	0.692	0.766	0.643	0.701	0.568	0.572
Logistic Regression	0.661	0.721	0.702	0.755	0.703	0.766	0.628	0.699	0.595	0.557
Perceptron	0.649	0.713	0.716	0.722	0.707	0.695	0.643	0.671	0.592	0.591
PLS DA	0.664	0.703	0.697	0.754	0.693	0.768	0.648	0.705	0.568	0.577
Random Forest	0.668	0.814	0.710	0.730	0.706	0.726	0.715	0.743	0.682	0.698
SGD	0.663	0.718	0.712	0.746	0.699	0.761	0.646	0.684	0.596	0.628
SVC	0.658	0.681	0.700	0.717	0.699	0.714	0.641	0.735	0.669	0.721
XGBoost	0.664	0.734	0.707	0.739	0.700	0.725	0.697	0.743	0.695	0.716

(B) Average Negative Predictive Value for the models for HNF1A-MODY

Table S6. Average Brier Score for each Dataset, two separate columns for the original unbalanced sample (unb), and with oversampling(ovr). The Brier Score ranges from 0 (perfect prediction) to 1 (completely inaccurate prediction)

	Zero		Half KNN		Half MICE		Full KNN		Full MICE	
**Model**	**unb**	**ovr**	**unb**	**ovr**	**unb**	**ovr**	**unb**	**ovr**	**unb**	**ovr**
GaussianNB	0.246	0.248	0.242	0.238	0.240	0.236	0.182	0.171	0.177	0.173
KNeighbors	0.242	0.232	0.237	0.236	0.226	0.226	0.192	0.197	0.171	0.182
LDA	0.220	0.227	0.211	0.223	0.205	0.219	0.188	0.232	0.174	0.216
Logistic Regression	0.221	0.236	0.215	0.231	0.208	0.223	0.188	0.234	0.174	0.224
Random Forest	0.228	0.231	0.212	0.221	0.199	0.204	0.158	0.174	0.150	0.168
XGBoost	0.235	0.258	0.250	0.266	0.216	0.232	0.168	0.194	0.165	0.187

(A) Average Brier Score for the models for GCK-MODY

	Zero		Half KNN		Half MICE		Full KNN		Full MICE	
**Model**	**unb**	**ovr**	**unb**	**ovr**	**unb**	**ovr**	**unb**	**ovr**	**unb**	**ovr**
GaussianNB	0.321	0.321	0.324	0.355	0.315	0.349	0.306	0.323	0.290	0.310
KNeighbors	0.255	0.241	0.213	0.312	0.229	0.307	0.249	0.281	0.259	0.271
LDA	0.313	0.302	0.222	0.270	0.218	0.265	0.235	0.240	0.256	0.267
Logistic Regression	0.253	0.251	0.211	0.258	0.209	0.252	0.241	0.239	0.242	0.260
Random Forest	0.218	0.175	0.220	0.228	0.226	0.238	0.211	0.216	0.234	0.238
XGBoost	0.234	0.241	0.221	0.260	0.218	0.269	0.223	0.227	0.228	0.247

(B) Average Brier Score for the models for HNF1A-MODY

Table S7. Optimal model hyperparameters for the machine learning strategy achieving the highest average ROC AUC score for each MODY subtype. The table summarizes the dataset configuration, machine learning technique used, and the best-performing hyperparameter values obtained during model optimization.

Dataset	Sub-dataset	ML Technique	Best Hyperparameters
GCK-MODY	Full MICE, oversampled	GaussianNB	`var_smoothing`: 10^{-11}
HNF1A-MODY	Full MICE, unbalanced	Random Forest	`max_depth`: 5<br>`max_features`: 2<br>`n_estimators`: 100
